# Cytoprotective, Antiproliferative, and Anti-Oxidant Potential of the Hydroethanolic Extract of *Fridericia chica* Leaves on Human Cancer Cell Lines Exposed to α- and β-Zearalenol

**DOI:** 10.3390/toxins15010036

**Published:** 2023-01-03

**Authors:** Neda Alvarez-Ortega, Karina Caballero-Gallardo, Cristina Juan, Ana Juan-Garcia, Jesus Olivero-Verbel

**Affiliations:** 1 Environmental and Computational Chemistry Group, School of Pharmaceutical Sciences, Zaragocilla Campus, University of Cartagena, Cartagena 130014, Colombia; 2 Functional Toxicology Group, School of Pharmaceutical Sciences, Zaragocilla Campus, University of Cartagena, Cartagena 130014, Colombia; 3 Laboratory of Food Chemistry and Toxicology, Faculty of Pharmacy, University of Valencia (Spain)—Avda, Vicent Andrés Estellés, s/n. Burjassot, 46100 València, Spain

**Keywords:** mycotoxins, ROS, cytotoxicity, plant extracts

## Abstract

*Fridericia chica* (Bignoniaceae) is a Colombian Caribbean plant with numerous health benefits, including properties such as wound healing, immune system stimulation, and antioxidant capacity, among others. Mycotoxins alpha-zearalenol (α-ZEL) and beta-zearalenol (β-ZEL) are phase I metabolites of zearalenone, a natural product involved in endocrine disruption and cell proliferation processes. This study aimed to investigate the cytotoxic potential of the hydroethanolic extract of *F. chica* leaves (HEFc) and determine their protective effects against proliferation induced by α-ZEL and β-ZEL on human hepatoma HepG2, lung cancer Calu-1, and primary normal human epidermal keratinocytes, neonatal (HEKn). The cytotoxicity of HEFc was measured in a range from 4 to 1000 µg/mL and from 0.4 to 100 μM for both α-ZEL and β-ZEL. Cell production of intracellular ROS was monitored using the H_2_-DCFDA probe. The cells exposed to HEFc presented IC_50_ of 128, 249, and 602 µg/mL for the HepG2, Calu-1, and HEKn cells, respectively. A greater selectivity was seen in HepG2 cells [selectivity index (SI) = 3.5] than in Calu-1 cells (SI = 2.4). Cells treated with mycotoxins remained viable during the first day, and cell proliferation increased at low tested concentrations (0.4-6.3 µM) in all three cell lines. However, after 48 h treatment, cells exposed to 50 and 100 µM of α-ZEL and β-ZEL displayed decreased viability. HEFc at 16 µg/mL was able to give some protection against cytotoxicity induced by high concentrations of β-ZEL in HepG2, reducing also cell proliferation elicited at low levels of α-ZEL and β-ZEL. ROS production was not observed in cells treated with this HEFc concentration; however, it prevented ROS formation induced by treatment with 50 µM α-ZEL or β-ZEL. In summary, HEFc isolated from plants grown in northern Colombia displayed promising results against cell proliferation and oxidative stress caused by mycotoxins.

## 1. Introduction

Medicinal plants have become a topic of global interest, as they are been used to treat various health problems in many countries [1]. In developing countries, many communities utilize phytomedicines as the first treatment when any sign or symptom appears. However, the study of the pharmacological potential of plants continues to be limited, but it is a relevant field to develop due to the possible production of new substances that improve human health. Only about 10% of medicinal plants have been analyzed to treat various diseases.

*Fridericia chica*, a shrub of the Bignoniaceae family, has several benefits for human health. This plant is commonly found in the humid tropical forests of Central America, Mexico, and the Amazon. Fermentation of its leaves produces a red dye that is frequently used by the natives to paint their bodies and utensils. Moreover, the infusion of this plant turns into a red liquid used in their traditional medicine as a pain killer [2], astringent, and as a treatment for diarrhea, anemia, and leukemia [1]. Recent studies have displayed the chemical complexity of the *F. chica* extract. Its activity has been reported as antimicrobial, antioxidant [3], anti-inflammatory [4], healing [5], leishmanicidal [6], and antigenotoxic [7].

Mycotoxins are small molecules produced by fungi, mainly by the genera *Penicillium* sp., *Aspergillus* sp., and *Fusarium* sp. [8]. Mycotoxins are one of the biotic factors that may appear in cereals before harvest and during storage. According to the FAO, the production of these foods is increasing, with an estimated production of 2799 million tons [9]. The European Food Safety Authority (EFSA) has estimated that approximately up to 80% of food products are contaminated with these toxins each year [10]. One of the most worrying mycotoxins is Zearalenone (ZEN). This mycotoxin is produced by *Fusarium* sp., and through different biotransformation pathways two different metabolites are generated, α-Zearalenol (α-ZEL) and β-Zearalenol (β-ZEL) [11]. The biotransformation of ZEN occurs in the liver, lung, kidney, and prostate, among other tissues of various animal species [12].

Mycotoxins are of special concern for human health [13]. Several studies have shown their occurrence and effects on human and animal health after exposure to ZEN metabolites [14,15,16,17], aflatoxin B1 [18], deoxynivalenol [19], and Ochratoxin A [19]. Hydroxyl derivatives, such as α-ZEL and β-ZEL, have endocrine disruption potential [17], and capacity to induce oxidative stress [20] and DNA damage [21], although according to the IARC, ZEN is a Group 3 carcinogen [22]. There are different in vitro studies that have shown its role in cell proliferation processes, including human hepatoma [23,24,25,26], endometrial adenocarcinoma [27], and breast cancer cells [28].

The present study investigated the antitumor potential of the hydroethanolic extract of *F. chica* leaves (HEFc) in lung (Calu-1) and liver cancer cells (HepG2) versus non-cancer skin cells (HEKn). In addition, cell viability was evaluated against mycotoxins α-ZEL and β-ZEL in these same cells, followed by the antiproliferative and anti ROS effect of the extract.

## 2. Results

### 2.1. Viability of HEFc on Cells Lines

**Figure 1:**
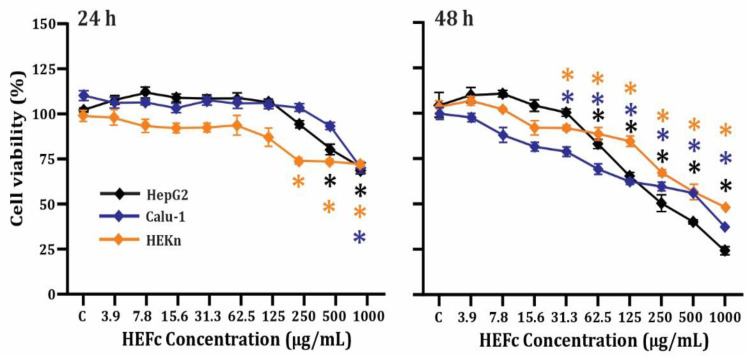
Cytotoxic activity of HEFc on cancerous (HepG2 and Calu-1) and non-cancerous (HEKn) cells after 24 h and 48 h exposure. Each result represents the mean ± standard error of the mean. * Significant difference (*p <* 0.05) when compared to the control group (C).

The results on the cytotoxicity of cancerous (HepG2 and Calu-1) and non-cancerous cells exposed to HEFc are shown in Figure 1. At the highest tested concentrations (500 and 1000 μg/mL) the extract exhibited cytotoxicity compared to the control (*p* < 0.05) during the first 24 h, but the IC_50_ value could not be determined for any of the three cell lines. However, the cytotoxic effect at 48 h showed a concentration-dependent trend, mainly in cancer cells. No increases in cell proliferation were observed in any case. In concordance with these results, the concentration of 16 μg/mL was selected for the cytoprotection assay. The selectivity index (SI) calculated for HEFc on cancer cell lines is presented in Table 1. The SIs for HEFc was greater than 1 for the two tumor cell lines. 

### 2.2. Viability of Cells Exposed to Mycotoxins

**Figure 2:**
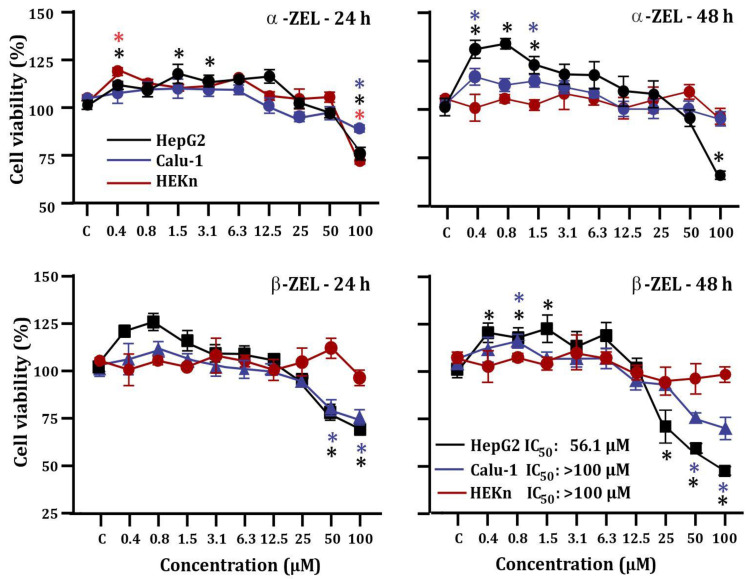
Cytotoxicity effects of mycotoxins (α-ZEL and β-ZEL) on HepG2, Calu-1, and HEKn cells after 24 h and 48 h exposure. Each result represents the mean ± standard error of the mean. * Significant difference (*p <* 0.05) when compared to the control group (C).

The viability of the cell lines exposed to mycotoxins after 24 h and 48 h of treatment are presented in Figure 2. A reduction in viability was observed in HepG2 cells exposed to 100 µM α-ZEL by approximately 25% and 50% after incubation for one and two days, respectively. Nonetheless, the IC_50_ could not be calculated. In contrast, the lowest tested concentrations (0.4, 0.8, and 1.5 μM) showed a significant increase in cell growth (*p* < 0.05). 

For β-ZEL, after 24 h and 48 h of exposure, cell viability was significantly reduced in the 50 µM and 100 µM treatments (*p* < 0.05) compared to the control. In contrast, after 48 h at 0.8 and 1.5 µM, cell proliferation significantly increased in HepG2 cells (*p* < 0.05). The IC_50_ was found in the HepG2 cell line after 48 h of exposure. Photographs of cell morphology changes in HepG2 cells exposed to 50 µM α-ZEL and β-ZEL after 24 h exposure are shown in Figure S1.

### 2.3. Antiproliferative Effects of HEFc against α-ZEL and β-ZEL

**Figure 3:**
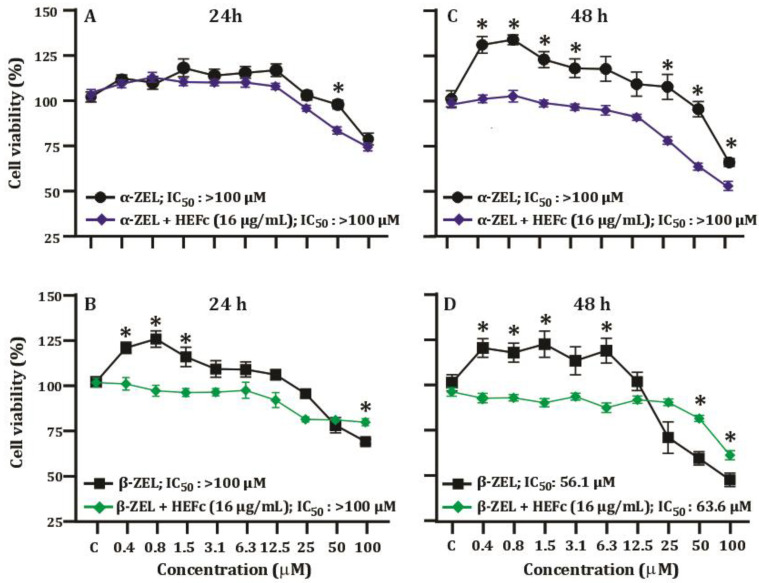
Anti-proliferative effects of HEFc on HepG2 cells exposed to α-ZEL or β-ZEL after treatment for 24 h (**A**,**B**) and 48 h (**C**,**D**). Each result represents the mean ± standard error of the mean. * Significant difference compared to the corresponding control group (C)*, p* < 0.05.

To develop the cytoprotection assay, HepG2 cells were selected since it was the only cell line with IC_50_ detection after 48 h of exposure (Figure 2). The cell proliferation induced by the mycotoxins α-ZEL and β-ZEL was markedly reduced in the presence of 16 µg/mL HEFc, after treatment for 24 or 48 h. In addition, the IC_50_ of the combination showed a considerable difference from that of β-ZEL alone, with a value of 63.6 μM (Figure 3).

### 2.4. ROS Levels of HepG2 Cells

**Figure 4:**
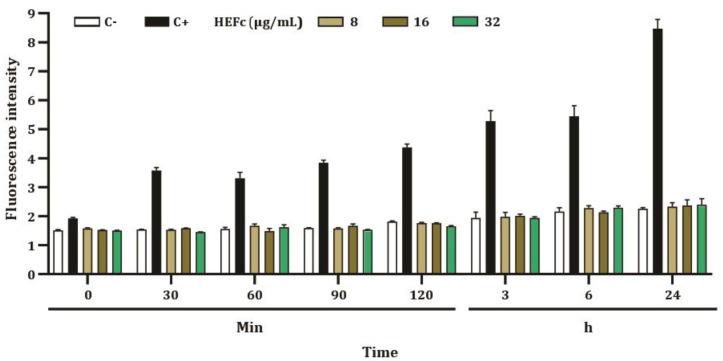
ROS levels of HepG2 cells exposed to HEFc (8, 16, and 32 µg/mL) after different exposure times. Each result represents the mean ± standard error of the mean. C- represents the negative control (DMSO 1%) and C+ the positive control (200 μM hydrogen peroxide).

**Figure 5:**
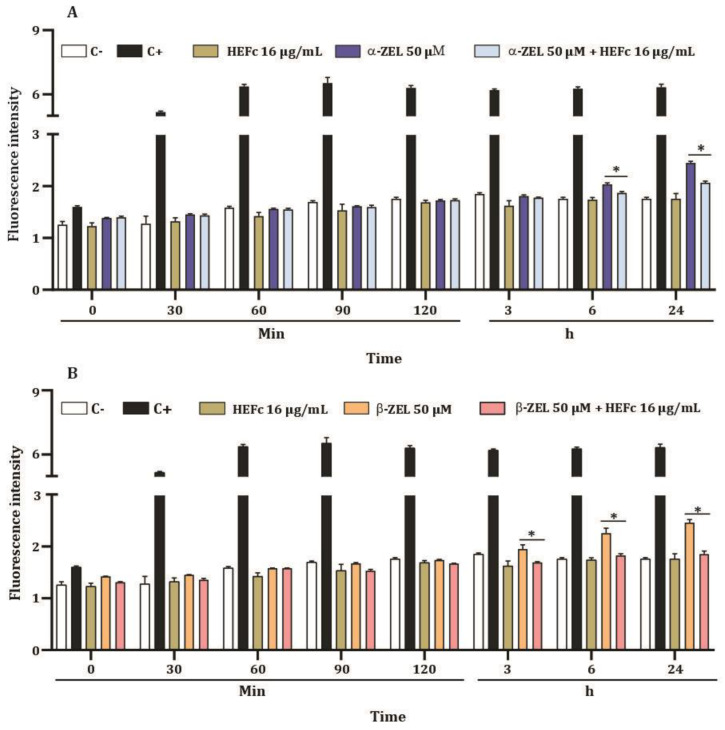
ROS levels of HepG2 cells. (**A**) ROS levels of HepG2 cells treated with HEFc (16 µg/mL), α-ZEL, or HEFc (16 µg/mL) + α -ZEL (50 µM). (**B**) ROS levels of HepG2 cells treated with HEFc (16 µg/mL), β-ZEL, or HEFc (16 µg/mL) + β-ZEL (50 µM). Negative control (C-), culture medium; positive control (C+), hydrogen peroxide (200 μM). * Significant differences (*p <* 0.05) compared to α-ZEL or β-ZEL alone.

The results of the ROS generation by HEFc (8, 16, and 32 μg/mL) on HepG2 cells are presented in Figure 4. DCF-DA fluorescence levels in cells treated with HEFc (8, 16, and 32 μg/mL) did not show significant changes when compared to the negative control. Afterwards, the anti-ROS effect of the extract (16 μg/mL) was evaluated. Incubation of HepG2 cells with 16 µg/mL HEFc for 6 h and 24 h reduced ROS generation induced by α-ZEL and β-ZEL (Figure 5).

## 3. Discussion

The increasing use of medicinal plants makes evaluating their toxicity a necessary step for developing new synthetic or natural pharmaceutical products. Toxicity tests are recommended to ensure the safe use of extracts obtained from medicinal plants. Due to the wide use of *F. chica* as a medicine for numerous health conditions, in this study, we developed tests to detect the SI of HEFc on hepatocarcinoma cells (HepG2) and lung cancer cells (Calu-1). The most potent effect on cell viability, with the lowest IC_50_ values, occurred in HepG2 cells. Based on these findings, we selected a concentration of 16 μg/mL of HEFc to perform bioassays on HepG2 with the combination mycotoxin-extract. Moreover, the HepG2 cell line was used due to its endogenous capacity for bioactivation, and their role on the metabolism of xenobiotics [29].

Cancer remains a main global health problem affecting both sexes [30]. This complex disease has diverse etiologies and multiple stages [31,32]. Its treatment is focused on cells with rapid division, although it affects normal cells [33]. Therefore, it is essential to search for bioactive molecules from various sources that can target specific molecular signaling pathways in tumor cells without damaging normal cells.

Extracts of *F. chica* have been used in traditional medicine as an anticancer agent against leukemia [34]. Furthermore, ethanolic and aqueous extracts of *F. chica* have immunomodulatory and antitumor activities on solid Ehrlich tumors, probably attributed to the presence of flavonoids. In the present study, the ethanolic extract of *F. chica* leaves exerted a more significant cytotoxic effect on cancer cells than on non-cancer cells. IC_50_ values were much lower in liver cancer cells than in the tested Calu-1. Previous studies with *F. chica* extracts, or its components, in cancer cells, such as Jurkat-1, HL60 [35] and SH5YSY cell lines [36] showed results consistent with our results. 

Zearalenone is an estrogenic mycotoxin whose role in carcinogenesis remains questionable. In this study, we evaluated the effect of exposure to ZEN metabolites on the proliferation of HepG2, Calu-1, and HEKn cells. Our results showed that the mycotoxins studied decreased cell viability at high concentrations, but stimulated cell proliferation at lower concentrations in HepG2 and Calu-1 cells. Similarly, this behavior has been reported for SH-SY5Y [37], MCF-7 [38,39], PALMA [40], MDA-MB-231, and T47D cells [13]. The cell proliferation exerted by ZEN and its metabolites may play a role in promoting hormone-dependent tumors because these compounds bind to and activate the ER (estrogen receptor) [37,41] due to their similar structure with the body’s natural hormone, 17-β-estradiol (E2). The finding in terms of proliferation reported here may be due to the expression of two ER (α-ER and β-ER), which has also been reported for other xenoestrogens acting on liver cancer cells [42]; however, signaling pathways are not yet fully established. It is plausible that micro-RNAs are also involved in stimulating cell proliferation by silencing various cell cycle inhibitors; accordingly, it has been stated that ZEN induces oxidative stress, which may be a regulator of miRNAs, interfering with micro-RNA expression [43].

Several pharmacological properties have been reported for HEFc, one of them has been the reduction of α-ZEL and β-ZEL-induced cell proliferation in SH5YSY [36]. The results presented here make possible to suggest that the antiproliferative activity of HEFc may be link to its flavonoid content, for instance, 6-hydroxyluteolin, which has shown good antiproliferative activity in cell lines such as MCF7, HepG2, and Caco2, among others [44]. The extract of *F. chica* is also a good source of Vicenin-2, a flavonoid with antioxidant, hepatoprotective, anti-inflammatory, and anticancer properties. Its action, as a potent inhibitor of cell proliferation, has been shown in colon cancer cells [45] and hepatocellular carcinoma [46]. Different components of *F. chica* have been tested for their antimicrobial action [47]. In addition to the properties mentioned, it should be noted that other authors have demonstrated the safety of the extract in an *in vivo* model with rats, where a dose greater than 200 mg/kg did not induce changes in biochemical and hematological parameters [47,48]. Similarly, Takenaka et al. [49] did not observe signs of toxicity after the treatment with 300 mg/Kg of hydroethanolic extract of *F. chica* in BALB/c mice.

In the present study, treatment with HEFc significantly protected cells from oxidative damage by inhibiting the generation of ROS induced by ZEN metabolites. This can be explained due to the leaves’ reported antioxidant properties given their chemical composition [50].

## 4. Conclusions

HEFc offers protection against the cytotoxic effects provoked by α-ZEL and β-ZEL on human liver and lung cancer cells. It also reduces ROS production on liver cancer cells. Further investigations are required to understand the mechanisms behind the hepatoprotective effect of *F. chica*.

## 5. Materials and Methods

### 5.1. Reagents

The reagents used were Eagle’s Minimum Essential Medium (EMEN) (Quality Biological, Gaithersburg, MD, USA); Fetal Bovine Serum (FBS) (Biowest, South America Origin); Keratinocyte Medium (KM) (ScienCell Research Laboratories, Carlsbad, CA, USA); dimethyl sulfoxide (DMSO), hydrogen peroxide 30% *v*/*v*, and phosphate-buffered saline (PBS) were supplied by Panreac (Applichem, Barcelona, Spain); α-ZEL and β-ZEL (MW: 320.38 g/mol) standards, penicillin, streptomycin, Trypsin–EDTA, and 2.7-dichlorofluorescein diacetate (DCFH-DA) were purchased from Sigma-Aldrich (St. Louis, MO, USA).

### 5.2. Extract Preparation and Characterization

Fallen leaves of *F. chica* samples were collected in Sincelejo at the University of Sucre (Sincelejo, Colombia). The species was identified by Pedro Alvarez Perez in the Herbarium at the University of Sucre (Sincelejo, Colombia). A voucher specimen was deposited in the Herbarium at the University of Sucre (Sincelejo, Colombia) under the number 004537. The hydroethanolic extract (ethanol:water; 7:3) preparation from leaves, as well as its characterization, were carried out as reported elsewhere [36]. The extract was reconstituted in DMSO and kept at −20 °C until use.

### 5.3. Maintaining Cell Culture

The HepG2 (ATCC-HB-8065) and Calu-1 cells were routinely cultured using EMEN, and supplemented with 10% FBS and 1% penicillin/streptomycin (Sigma-Aldrich, St. Louis, MI, USA) as previously described [45,51]. Additionally, 5 × 10^5^ primary HEKn cells were thawed in KM culture medium (ScienCell Research Laboratories, Carlsbad, CA, USA) supplemented with KGS 100× (ScienCell Research Laboratories, Carlsbad, CA, USA) and 1% penicillin/streptomycin solution (ScienCell Research Laboratories, Carlsbad, CA, USA). After cells reached ~80–90% confluency, they were washed with DPBS and trypsinized to detach from the culture flask. Trypsin-EDTA 0.025% (2 mL) was added to the monolayer and incubated for 3–5 min, and once detached, 2 mL of TNS was added to the cell suspension and centrifuged at 1200 rpm for 3 min. The supernatant was discarded, and the pellet was resuspended in growth medium and replaced in a 1:4 ratio. Passages 2–4 were used for the experiments. All cell lines were grown at 37 °C in 5% CO_2_.

### 5.4. Exposure of HepG2, Calu-1, and HEKn Cells to F. chica Extract and Mycotoxins

The cell lines were cultured in 96-well plates at a density of 2 × 10^4^  cells/well for 24 h. Then, the cells were treated at 24 h and 48 h with 100 μL of fresh medium containing different concentrations of (a) HEFc (from 1000 to 3.9 µg/mL, 1:2 dilutions), (b) α-ZEL (from 100 to 0.4 μM, 1:2 dilutions), or (c) β-ZEL (from 100 to 0.4 μM, 1:2 dilutions). These concentrations have been reported previously. The 1% DMSO vehicle was used as a control. After exposure to mycotoxins or extracts, the viability assay was performed using MTT in the same way as reported in our previous study [46,52]. Optical density was measured on a spectrophotometer Varioskan™ LUX (Thermo Fisher Scientific, Inc., Waltham, MA, USA) at 620 nm. The experiments were developed three times with four replicates each. Viability was defined as the ratio (expressed as a percentage) of the absorbance of treated cells to control cells.

### 5.5. Cytoprotective Effects of HEFc against ZEN Metabolites

To determine the cytoprotective effect of HEFc on mycotoxin-induced toxicity, the working concentration of the extract was selected considering the largest concentration at which no significant cytotoxicity was observed (16 µg/mL). Two independent treatment combinations were carried out. They consisted of a mixture of HEFc at 16 µg/mL (fixed concentration) with eight concentrations of α-ZEL or β-ZEL (from 0.4 to 100 µM, 1:2 dilutions). The plates were incubated for 24 h and 48 h at 37 °C in a 5% CO_2_ atmosphere, and viability was examined using the MTT assay. Three experiments were carried out with four replicates each. The selectivity index (SI) was obtained using the ratio of the IC_50_ of non-cancer cells versus the IC_50_ of cancer cells.

### 5.6. ROS Assay

Intracellular ROS levels were monitored in HepG2 cells using the 2,7-dichloro-fluorescein diacetate probe (DCFH-DA; Sigma–Aldrich, St. Louis, MO, USA). Briefly, 2 × 10^6^ cells/plate were seeded in a 96-well black culture microplate. After incubation for 24 h, the culture medium was removed and supplemented, and EMEN containing 20 μM of DCFH-DA probe was added for 20 min under the same incubation conditions [47]. Later, the cells were washed with PBS (Panreac Applichem^®^, Barcelona, Spain) and exposed to HEFc (16 μg/mL). A solution of 200 μM H_2_O_2_ (Panreac Applichem^®^, Barcelona, Spain) was used as a positive control. The increase in fluorescence was measured for 120 min after 3, 6, and 24 hours of exposure using a Varioskan TM LUX Multimode Microplate Reader (Thermo Fisher Scientific, Inc., Waltham, MA, USA). The fluorescence intensity was determined by employing 485 nm for excitation and 530 nm for emission. The results were expressed as the fluorescence values normalized over the control (untreated cells). Three independent experiments were performed with three replicates each.

### 5.7. Statistical Analysis

Cell viability data are presented as mean±SEM, and normality was assessed using Shapiro-Wilk test. Statistical comparisons between means were carried out using one-way analysis of variance (ANOVA), followed by Dunnett’s test. IC_50_ values for each treatment were obtained utilizing nonlinear sigmoid curve fitting. All results were processed employing GraphPad 5.0 Instat Software (San Diego, CA, USA). The results were considered significant at *p* < 0.05.

## Figures and Tables

**Table 1:** Selectivity index (SI) of HEFc on HEKn relative to cancer cell lines (HepG2 and Calu-1).

	IC_50_ (48 h)	SI
HepG2	168	3.5
Calu-1	249	2.4
HEKn	602.9	-

## Data Availability

The datasets used and analyzed during the current study are available from the corresponding author on reasonable request.

## Supplementary Materials

The following supporting information can be downloaded at: https://www.mdpi.com/article/10.3390/toxins15010036/s1, Figure S1: Morphology of HepG2 cells after 24 h incubation. Photographs taken under phase contrast. Control (1% DMSO). Scale bar: 200 µm.
