# Supplementary material for: Cytoprotective, Antiproliferative, and Anti-Oxidant Potential of the Hydroethanolic Extract of Fridericia chica Leaves on Human Cancer Cell Lines Exposed to α- and β-Zearalenol
Source: Toxins (Basel). 2023 Jan 3;15(1):36. doi: 10.3390/toxins15010036 (PMC9864583; doi:10.3390/toxins15010036)
Supplement: Supplementary file 1 [file toxins-15-00036-s001.zip › toxins-2018871-supplementary.pdf]

Supplementary materials: Cytoprotective, antiproliferative, and anti-oxidant potential of the hydroethanolic extract of *Fridericia chica* leaves on human cancer cell lines exposed to α- and β-Zearalenol

Neda Alvarez-Ortega, Karina Caballero-Gallardo, Cristina Juan, Ana Juan-Garcia, Jesus Olivero-Verbel

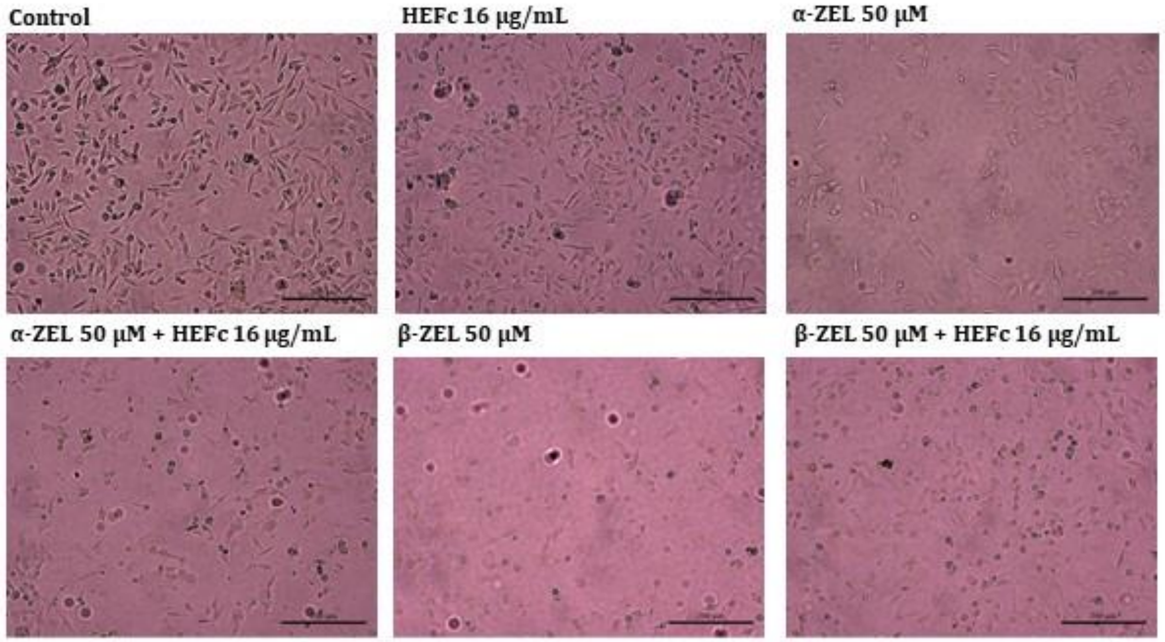

Figure S1. Morphology of HepG2 cells after 24 h incubation. Photographs taken under phase contrast. Control (1% DMSO). Scale bar: 200 μm.
